# Rapid transcellular hepatic copper depletion by ARBM-101 rescues severe liver damage in Wilson disease rodents

**DOI:** 10.1016/j.biopha.2025.118867

**Published:** 2025-12-04

**Authors:** Jonas Engler, Eun-Jung Kim, Dasol Kim, Noreene M. Shibata, Emilie Munk Lynderup, Mikkel H. Vendelbo, Banu Akdogan, Judith Sailer, Adriana Fontes, Carola Eberhagen, Tamara Rieder, Quirin Reinold, Hongjae Lee, Dongsik Park, Chunwon Jung, Weonbin Im, Susanne I. Wudy, Karin Kleigrewe, Stefan Engelhardt, Alan A. DiSpirito, Thomas Damgaard Sandahl, Valentina Medici, So-Young Eun, Hans Zischka

**Affiliations:** a Institute of Toxicology and Environmental Hygiene, TUM School of Medicine and Health, Technical University of Munich, Munich, Germany; b R&D Center, ArborMed Co. Ltd, Pangyo, Seongnam, Gyeonggi-do, Republic of Korea; c Department of Internal Medicine, Division of Gastroenterology and Hepatology, University of California Davis, Davis, CA, USA; d Department of Hepatology and Gastroenterology, Aarhus University Hospital, Aarhus, Denmark; e Department of Nuclear Medicine and PET Center, Aarhus University Hospital, Aarhus, Denmark; f Department of Biomedicine, Aarhus University, Aarhus, Denmark; g Institute of Molecular Toxicology and Pharmacology, Helmholtz Munich, German Research Center for Environmental Health, Neuherberg, Germany; h Bavarian Center for Biomolecular Mass Spectrometry (BayBioMS), TUM School of Life Sciences, Technical University of Munich, Freising, Germany; i Institute of Pharmacology and Toxicology, TUM School of Medicine and Health, Technical University of Munich, Munich, Germany; j Roy J. Carver Department of Biochemistry, Biophysics and Molecular Biology, Iowa State University, Ames, IA, USA

**Keywords:** Wilson disease, Methanobactin, Copper excretion, Liver, Mitochondria

## Abstract

In Wilson disease (WD), excess copper provokes hepatocyte death due to impaired copper excretion, ultimately causing either acute or chronic liver damage. Current therapeutic compounds fail to reduce hepatic copper near to physiological levels, leaving lifelong, several times daily treatment as the only choice for patients. We have previously shown that a bacteria-derived methanobactin, termed ARBM-101, most efficiently depleted excess liver copper in still healthy WD rats. Here we report, for the first time, that mechanistically this is due to endosomal/lysosomal/exosomal trafficking of ARBM-101 in WD hepatocytes, allowing for copper mass excretion via the biliary/fecal route. We further show that such liver copper excretion occurs within minutes *in vivo* to detect copper-bound ARBM-101 in feces. This efficacy allows for specialized treatment regimen to rescue acute liver failure in WD rats. Moreover, also shown for the first time, it avoids fibrosis development in WD mice. Thus, judging from the results in two rodent species and human hepatocytes, this study advocates the development of ARBM-101 for WD therapy.

## 1. Introduction

Wilson’s disease (WD) is a rare autosomal recessive disorder of copper metabolism caused by mutations in the *ATP7B* gene, which encodes a copper-transporting ATPase primarily expressed in hepatocytes. The defect impairs biliary copper excretion from the liver, leading to progressive accumulation of toxic copper in the liver, brain, and other organs [1–5]. Untreated, WD is uniformly fatal. In the liver, hepatic copper overload results in hepatocellular injury, manifesting clinically as inflammation and liver injury, chronic progressive liver disease, or, in severe cases, acute liver failure [6]. The estimated WD prevalence is approximately 1 in 30,000 individuals [7].

We and others have previously reported that, in WD, excess copper proteo-toxically impairs hepatocyte mitochondria causing progressive structural and functional deficits that pave the way towards cellular demise [8–11]. Hepatocytes aim to counterbalance this by enforced auto/mitophagy thereby affecting the lysosomal compartment with unbalanced copper load as well [8,12]. While this cellular recycling machinery may delay severe consequences, it will ultimately fail due to the continuous copper uptake via nutrition [12]. As a steadily increasing massive copper amount progressively impairs hepatic mitochondria and lysosomes, we thus aimed for a compound-driven copper mass transportation and systemic depletion as remedy [8,11].

Methanobactins, bacteria-originated chalkophores [13,14], are highly potent copper chelators uniquely capable of reducing liver copper in preclinical models of WD [11,15,16]. In still healthy WD rats, eight days of treatment reduced liver copper levels close to wildtype levels [11]. Importantly, a dose-dependent liver copper reduction using the methanobactin isoform SB2 (ARBM-101), resulted, upon treatment stop, in a concomitantly delayed hepatitis onset and animal death [11,17]. These results demonstrated that liver failure and death are directly linked to the absolute copper amount in these animals.

We therefore asked how ARBM-101, mechanistically, resulted in such rapid and unprecedented liver copper depletion and found, in a human WD hepatocyte model, the transcellular trafficking of ARBM-101 via the endosomal/lysosomal/exosomal axis. This routing would allow for copper mass export via the biliary/fecal route that was found to be the case *in vivo*. Most importantly, such rapidity of fecal Cu- ARBM-101 excretion allows for greatly intensified treatment regimen that rescues from acute liver failure, even in severely diseased female WD rats, but also avoids the development of fibrosis in WD mice.

## 2. Materials and methods

### 2.1. Copper chelators and chemicals

Methanobactin (MB-SB2, recently termed ARBM-101) was isolated from *Methylocystis* sp. SB2 as previously described [11,13,18]. Doses were formulated in either PBS or 0.9 % NaCl and 0.2 μm sterile filtered for each administration. D-PA was a gift from Firma Heyl Pharma or purchased (Sigma P4875, St. Louis, MO).

### 2.2. WD mice

The *Atp7b*^−/−^ global knockout mouse model on a B6 background was kindly provided by Dr. Svetlana Lutsenko at Johns Hopkins University. *Atp7b*^−/−^ (WD) mice were maintained by a heterozygous breeding colony at University of California Davis (USA) animal facility to generate both homozygous mutant and wildtype controls. Mice were housed in standard polycarbonate cages with Teklad TEK-Fresh bedding (Envigo, Madison, WI) and nesting enrichment material under the following conditions: 22 ± 1 °C temperature, 50 ± 20 % relative humidity, 14-h light/10-h dark light-cycle, *ad libitum* LabDiet 5001 (13 mg/kg copper; PMI, St. Louis, MO) and deionized water. Mice were group-housed up to 3 per cage in treatment phase or single-housed in observation phase.

### 2.3. WD rats

The *Atp7b*^−/−^ rat strain (LPP rats), originally provided by Jimo Borjigin [19], was bred at the Helmholtz Munich (Germany) animal facility or at Aarhus University Hospital (Denmark). Animals were on a 12-h light/dark cycle. Temperature and relative humidity were 22 ± 1 °C and 50 ± 5 %, respectively, according to the European Convention 2007/526 EC. Animals were maintained *ad libitum* on the Altromin diet 1314 (13 mg/kg copper) and treated under the guidelines for the care and use of laboratory animals of Helmholtz Munich. Heterozygous *Atp7b*^+/−^ or homozygous *Atp7b*^+/+^ rats served as controls in this study.

### 2.4. Animal experiments

Mouse husbandry and environmental conditions followed UC Davis IACUC (protocol # 22563) and AAALAC regulations. Rat experiments were done in accordance with the EU Animal Welfare Act and approved by the government authorities on animal care (Germany: permit No: 55.2–2532.Vet_02–22–68 from the Government of Oberbayern; Denmark: permit No: 2021–15–0201–01069 from the Danish Animal Experiments Inspectorate).

### 2.5. In vivo treatments

*Atp7b*^−/−^ mice were intraperitoneally injected once every other day with freshly dissolved ARBM-101 (molar mass of 851.2 g/mol) for two consecutive weeks at 200 mg/kg bw (i.e. 235 μmol/kg bw, termed “intense treatment”) starting at an age of 84 days (Fig. 6A). This treatment period was followed by an observation period where the 200 mg/kg dose was provided i.p. every 2 weeks from the end of the treatment period to the end of the study for a total of 6 injections (at 28, 42, 56, 70, 84, 98 days of study). D-PA (molar mass of 149.21 g/mol) was provided to a separate group of *Atp7b*^−/−^ mice orally in drinking water at 100 mg/kg bw (i.e. 670 μmol/kg bw) for 14 days of study (treatment period) or from day 0–98 of the study (observation period). Urine and feces were collected over a 24-hour period before treatment start, at intense treatment end after 14 days (EOIT, Fig. 6A) and at study end (98 days, EOS, Fig. 6A) to analyze copper excretion. Body weight and food/water intake were monitored for adverse effects and to determine if dosage volumes needed to be adjusted over time.

*Atp7b*^−/−^ rats were intraperitoneally injected with ARBM-101 twice or thrice daily at 110 mg/kg bw (i.e. 130 μmol/kg bw), or D-PA once daily at 100 mg/kg bw (i.e. 670 μmol/kg bw) (Fig. 3A). Urine and feces were collected over a 24-hour period before treatment starts and at treatment end, to analyze copper excretion. Body weight and food/water intake were monitored for adverse effects.

### 2.6. Metabolic profiling of animals

Mice were placed in cages with a wire screen standing above LabSand (Coastline Global, Inc., West Chester, PA) instead of contact bedding. Food-in and water-in were weighed. A plastic mouse house was provided as a solid-surface option for mice to stand upon. After 24 h, mice were placed back into their normal contact-bedding cages. Food-out, water-out, and spill were weighed, and eat/day (g) and drink/day (mL) were calculated. Feces and urine were collected from the surface of the LabSand, measured by weight (feces) or pipette volume (urine), and stored at −75 °C until metal analysis.

Rats were housed individually in metabolic cages (Tecniplast^™^ Germany) for 24 h before and after treatment. Body weight change and food consumption were monitored. Urine and feces of each rat were collected. Feces were separated from chow residues, and urine samples were cleared by centrifugation for subsequent metal content determinations.

### 2.7. Histological examination

Mouse liver tissues were fixed in 10 % zinc formalin and embedded in paraffin. Liver samples were sectioned and stained with H&E or Sirius Red for analysis. Mouse grading and staging was done as previously published [20]. Lobular and portal inflammation were graded on a 4-point scale based on inflammatory foci within the liver section: grade 0 (none), 1 with 1–2 foci, 2 with 3–4 foci, 3 with > 4 foci. Fibrosis was graded 0 (none), 1 (expansion of portal fibrous tissue), 2 (early bridging, no nodules), 3 (bridging fibrosis, early nodule formation), and 4 (cirrhosis). Percent positive Sirius Red staining was obtained by sampling a 1 × 1 mm tile from each slide then building an algorithm to recognize Sirius Red from background. The algorithm was then applied to the entire tissue section on each slide, with each section divided into 1 × 1 mm tiles to cover the entire section. A similar method was used for nuclear sizing except tissue sections were divided into 0.5 × 0.5 mm tiles, with the algorithm recognizing hepatocyte nuclei (excluding lymphocytic nuclei and broken nuclear structures). All mouse histology and evaluations were performed by the UC Davis Center for Genomic Pathology Laboratory.

Rat liver tissues were fixed overnight at 4 °C in 4 % paraformaldehyde and embedded in paraffin. Tissue samples were cut into 4-mm-thick sections and stained with H&E for analyses. Embedding and staining were performed by the Institute for General Pathology and Pathological Anatomy at Technical University Munich.

### 2.8. Blood count, AST, ALT, ALP and T-bilirubin plasma-level

For mice, blood samples for complete blood count (CBC) analysis were collected in Sarstedt K3EDTA tubes and placed in a pre-cooled tube rack on ice. Blood samples for liver/kidney serology were collected in Sarstedt serum separator tubes, sat at room temperature (RT) for 30–45 min, centrifuged at 10,000 × g for 5 min, and serum collected; no hemolysis was observed. EDTA whole blood and serum samples were analyzed at the UC Davis Comparative Pathology Lab.

For rats, blood samples were taken sublingually. Heparin plasma AST, ALT, and T-bilirubin were determined with a Reflotron system (Roche). Heparin plasma from the vena cava was used for metal analysis.

### 2.9. ^64^Cu-positron emission tomography

*Atp7b*^−/−^ rats were intravenously injected with ^64^CuCl_2_ (^64^Cu) or ^64^Cu prebound to ARBM-101. Positron Emission Tomography/Magnetic Resonance Imaging (PET/MRI) scanning was performed for 20 min starting 60 min after the injection (t = 60 to t = 80) to visualize the ^64^Cu/^64^Cu-ARBM-101 distribution. Image requisition and PET data handling have been described in detail elsewhere [21]. Briefly, PET images were reconstructed with a three-dimensional ordered subset expectation algorithm with four iterations, six subsets, and a voxel size of 0.6 × 0.6 × 0.6 mm^3^.

### 2.10. Mitochondrial and lysosomal enriched fractions

Rat liver mitochondria and lysosomal enriched fractions were isolated from freshly prepared liver homogenates, as described previously [10,22,23] and purified by differential and density gradient centrifugation using Nycodenz^®^ (Axis-Shield).

### 2.11. Copper quantification

Mouse urine samples were centrifuged for 10 min at 9,000 × g and 4 °C to clear debris and a 200 μL aliquot used for analysis. Entire mouse fecal samples were used for analysis. Mouse liver samples were taken from the median lobe (100 ± 10 mg). All samples were digested with HNO_3_ (50 %; A509P500 Fisher Scientific) / H_2_O_2_ (6 %; NC1199178 Fisher Scientific) matrix in a 65 °C water bath, then diluted with milli-Q water to a total volume of 10 mL. ICP-MS was performed on a Thermo iCapQ ICP-MS (Thermo Fisher Scientific, Waltham, MA, USA) operating in KED mode and equipped with an ESI SC-2DX PrepFAST autosampler (Omaha, NE, USA). Samples were analyzed at the Northwestern University Quantitative Bio-element Imaging Center.

Rat samples were analyzed by inductively coupled plasma optical emission spectroscopy (Ciros Vision, SPECTRO Analytical Instruments GmbH) after sample treatment with 65 % nitric acid, as described previously [8].

### 2.12. Electron microscopic analysis

Electron microscopic analysis of liver tissues was done as previously described [24].

### 2.13. LC-MS/MS analysis

For studying metabolization of ARBM-101, the peptide was incubated with liver homogenate from WT and WD rats for up to 1 h and samples were taken every 15 min. 50 μL of the liver homogenate was diluted with 150 μL methanol. After mixing for 1 min, the samples were centrifuged (10 min, 10 °C and 13,000 rpm), and the clear supernatant was used for LC-MS/MS analysis. For LC-MS/MS analysis of feces, 50 mg of sample was added to 200 μL of methanol and mixed for one minute. The mixture was centrifuged (10 min, 10 °C and 13,000 rpm), and the supernatant was taken for LC-MS/MS analysis. Untargeted LC-MS/MS was done in a Nexera UHPLC system (Shimadzu, Duisburg, Germany) coupled with a Q-TOF mass spectrometer (TripleTOF 6600, AB Sciex, Darmstadt, Germany). Separation of ARBM-101 and Cu-bound ARBM-101 was achieved using an UPLC ACQUITY Premier BEH C18 2.1 × 100 mm, 1.7 μm analytical column (Waters, Eschborn, Germany) with a flow rate of 300 μL/min. The mobile phase consisted of 25 mM ammonium acetate in water (eluent A), and methanol (eluent B). The gradient profile was: 1 % B from 0 to 0.5 min, increasing to 99 % B at 2.5 min, and holding for 0.5 min. A volume of 2 μL of the sample was injected. The autosampler was cooled to 10 °C, and the column oven was heated to 40 °C. The samples were analyzed in the Information Dependent Acquisition (IDA) mode in positive electrospray ionization (ESI) mode. MS settings were as follows: Gas 1, 55; Gas 2, 65; Curtain gas, 35; Temperature, 500 °C; Ion Spray Voltage, 5500; declustering potential, 80. The mass range of the TOF MS and MS/MS scans was 100–1500 *m/z*, and the collision energy was set to 35 V with a 15 V spread. Ion chromatograms for the respective precursors ARBM-101 and Cu-bound ARBM-101 were extracted using PeakView (AB Sciex, Darmstadt, Germany). For metabolite annotation, the exact masses and MS2 fragmentation patterns of the most abundant signals were annotated using LibraryView (AB Sciex, Darmstadt, Germany).

### 2.14. RNA isolation and quantitative PCR

RNA was isolated from 25 mg mouse liver using the RNeasy Plus Mini kit (QIAGEN, Valencia, CA) according to the manufacturer’s instructions. RNA purity, concentration, and integrity were assessed by NanoDrop spectrophotometer (ThermoFisher Scientific, Waltham, MA) and agarose gel electrophoresis. The Superscript III First-strand Synthesis System (Invitrogen, Carlsbad, CA) was utilized to reverse-transcribe 5 μg RNA into cDNA. Quantitative PCR was run on a ViiA 7 Real-Time PCR System (Applied Biosystems, Foster City, CA) using SYBR Green master mix (Applied Biosystems, Foster City, CA) and a 1/25 cDNA dilution plated in triplicate along with a no-template control. Relative expression was calculated using 2-ΔΔCt with WT control as the calibrator. *Ndufs3* was used as a reference gene. Primer sequences for *Col1a1*, *Mt1*, and *Mt2* are listed in the table below (Table 1).

### 2.15. Cell culture treatment

Hepatocyte carcinoma HepG2 cells (ATCC, HB-8065) were maintained in 5 % CO_2_ at 37 °C and cultured in MEM media (Gibco, 11095098) supplemented with 10 % (v/v) fetal bovine serum (Gibco, 16000044) and 1 % (v/v) penicillin/streptomycin (Gibco, 15140122). ARBM-101 was freshly dissolved in distilled water prior to each experiment. For the preparation of 12.68 mM histidine-Cu (His-Cu), 0.216 g of Cu(II)chloride dihydrate (Sigma Aldrich, 459097) and 0.733 g of L-histidine (Sigma Aldrich, H6034) were dissolved in 100 mL 0.9 % NaCl buffer (pH 7.4). The solution was passed through a 0.2 μm syringe filter and stored at 4 °C. For 50 mM copper-prebound ARBM-101 solution, 100 mM CuCl_2_ and 100 mM ARBM-101 were mixed in equal volumes and incubated for 1 h at RT to make copper-prebound ARBM-101. In case of copper-free ARBM-101 treatment, cells were pretreated with 0.5 mM His-Cu for 24 h prior to ARBM-101 treatment.

### 2.16. Knock-down (KD) with siRNA transfection

The siRNAs were diluted to 10 μM and transfected into cells at 20 pmol for the individual siRNAs using Lipofectamine RNAiMAX transfection reagents (Thermo, 13778075) according to manufacturer’s instructions. Briefly, 2 × 10^4^ HepG2 cells were seeded onto each well in 96-well plate (SPL, 30006) and incubated overnight. Cells were treated with siRNAs for 24 h, and KD efficiency was validated by immunoblotting using anti-ATP7B mAb (Abcam, ab131208), anti-VAMP3 (Cell Signaling Technology, 13640), and anti-Aldolase (Cell Signaling Technology, 3188 s) antibodies. The following siRNAs were used: siControl (Thermo, 4390843), ATP7B siRNA (Thermo, s1821); VAMP3 siRNA (Horizon, L-011934–00–0005).

### 2.17. Immunocytochemistry (ICC)

HepG2 cells were plated onto the microscope cover glasses (Marienfeld, 0111520) in a 24-well plate. Upon treatments, cells were washed three times with PBS and fixed with 4 % formaldehyde in PBS for 10 min at RT. Cells were permeabilized with 0.5 % Triton X-100 in PBS for 5 min at RT. Blocking buffer (1 % BSA in PBS) was used for 30 min at RT. ARBM-101 was detected using an in-house produced monoclonal human Fc chimeric rabbit antibody at 2 μg/mL diluted in PBS overnight at 4 °C. Cells were washed three times with PBS and incubated with the secondary antibody Alexa Fluor^™^ 647-conjugated AffiniPure goat anti-human IgG (Jackson ImmunoResearch, 109–605–003) and Alexa Fluor^™^ 594 Phalloidin (Invitrogen, A12381) for 1 h at RT. Hoechst 33342 (Invitrogen, H3570) was applied for 10 min and washed with PBS. Fluorescent intensities were acquired under the confocal microscope (Carl Zeiss AG, Zeiss LSM 900) and analyzed by ImageJ software.

### 2.18. Endocytosis

For the macropinocytosis test, cells were pre-incubated with 100 μM EIPA (5-[N-ethyl-N-isopropyl] amiloride), an inhibitor for macropinocytosis, for 30 min, and subsequently treated with 1 mM ARBM-101 for further 30 min at 37 °C. For clathrin-mediated endocytosis (CME) test, 10 μg/mL chlorpromazine and 150 μM dynasore were used in the media for 30 min at 37 °C and then ARBM-101 was added to the media for 30 min at 37 °C. Cells were analyzed by confocal microscopy.

### 2.19. Exocytosis

WT and *VAMP3* KD HepG2 cells were incubated with 1 mM ARBM-101 for 1 h to observe the internalization of ARBM-101. To check the excretion of ARBM-101 by exocytosis, treated cells were changed to fresh media for a further 1 h to eliminate extracellular ARBM-101. The levels of intracellular ARBM-101 were compared between WT and VAMP3-depleted HepG2 by confocal microscopy as described above.

### 2.20. HepG2 couplet formation

HepG2 couplets were essentially generated *in vitro* as previously described [25–27]. Briefly, to generate extracellular matrix (ECM), 3 × 10^4^ HepG2 cells were plated onto the microscope cover glasses in 24-well plates for 3 days and incubated in autoclaved water for 45 min at 37 °C. Residual cells were removed by pipetting and the cover glasses were further incubated in MEM media for 1 h at 37 °C. 1 × 10^4^ HepG2 WT cells were re-plated onto the glasses for 24 h and siRNA for ATP7B and 0.5 mM His-Cu were applied for further 2 days. Cells were incubated with 1 mM ARBM-101 in fresh media for 15 min. Anti-ARBM-101 (R1) monoclonal antibody and anti-MRP2 antibody (Cell Signaling Technology, 4446) treatment was done overnight at 4 °C. As secondary antibodies, Alexa Fluor^™^ 647 goat anti-human IgG and Alexa Fluor^™^ 488 goat anti-rabbit IgG (Invitrogen, A11008) were used for ARBM-101 and MRP2, respectively. Alexa Fluor^™^ 594 Phalloidin was applied for 1 h, and Hoechst 33342 for 10 min at RT and washed with PBS. Cells were analyzed by confocal microscopy.

### 2.21. Intracellular localization of ARBM-101

To visualize the localization of ARBM-101, WT and *VAMP3* KD HepG2 cells were incubated with 1 mM ARBM-101 for 1 h. Upon media change, cells were incubated for the indicated time and fixed with 4 % formaldehyde. Cells were incubated with primary antibodies for EEA1 (Cell Signaling Technology, 3288), LAMP1 (Cell Signaling Technology, 15665), or CD63 (Santa Cruz, sc5275), along with R1 antibody overnight at 4 °C. Secondary antibodies were applied as follows: Alexa Fluor^™^ 488 goat anti-rabbit IgG for EEA1, Alexa Fluor^™^ 488 goat anti-mouse IgG (Invitrogen, A11001) for LAMP1, and Alexa Fluor^™^ 488 goat anti-mouse IgG for CD63. For the R1 primary antibody, Alexa Fluor^™^ 647 goat anti-human IgG was applied, and Alexa Fluor^™^ 594 Phalloidin was used to stain the cell membrane. Cells were analyzed based on the method for ICC and observed by confocal microscopy.

### 2.22. Statistics

Individual and mean data with standard deviation are given in the Figures. If the data set consisted of more than 2 replicates, the data sets were first tested for normality distribution and then parametric or nonparametric tests accordingly chosen. Correction for multiple testing was done. Statistical analyses were performed using GraphPad Prism 10 (GraphPad Software Inc).

## 3. Results

### 3.1. Transcellular hepatocyte routing of ARBM-101 via the endosomal/exosomal pathway

**Fig. 1.**
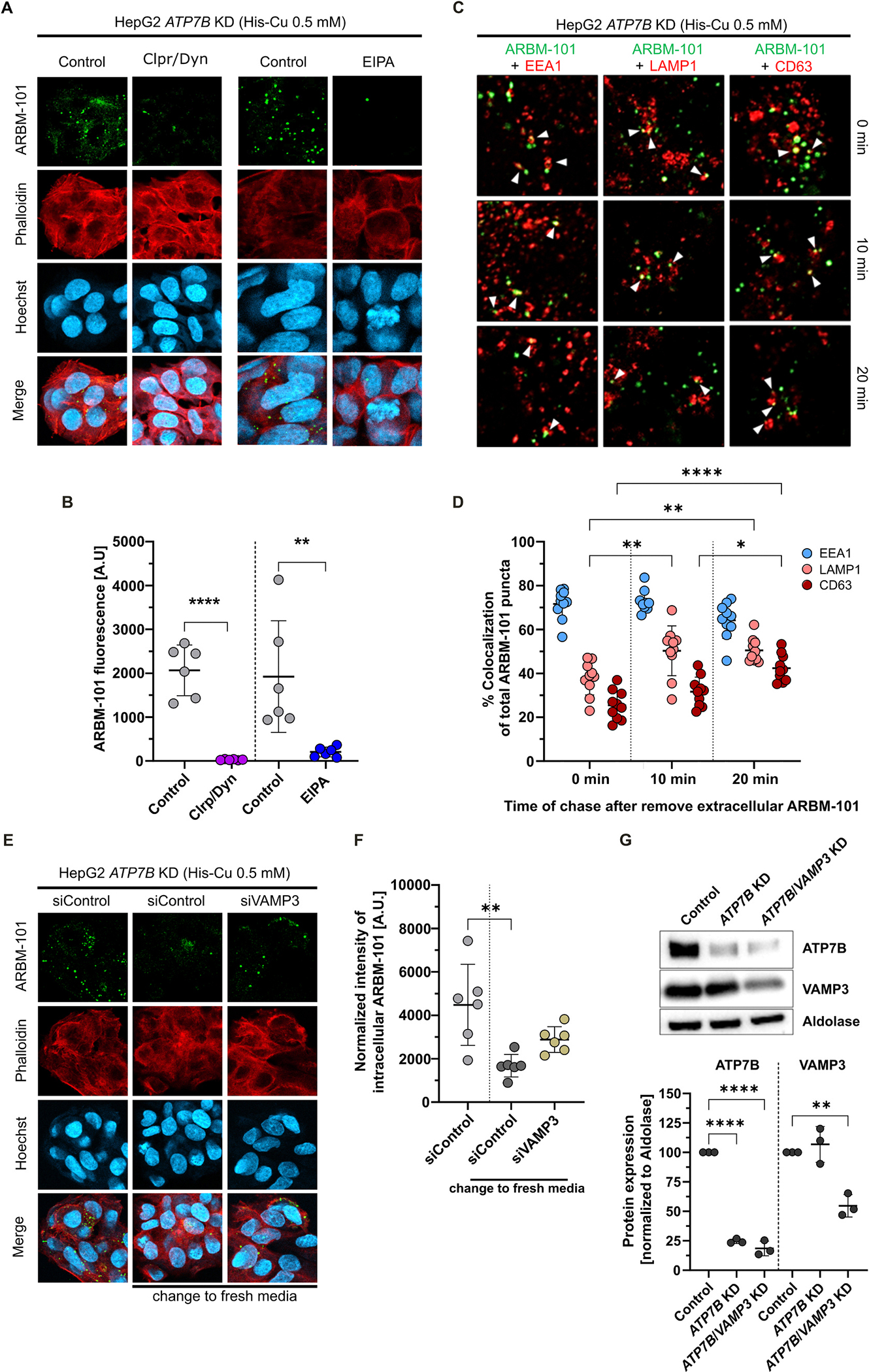
Transcellular hepatocyte routing of ARBM-101 via the endosomal/exosomal pathway. Representative confocal images of macropinocytosis and CME of ARBM-101 in *ATP7B* KD HepG2 cells (A). The intracellular fluorescent signal of ARBM-101 was detected in untreated control cells, EIPA-treated cells and Chlorpromazine-Dynasore(Clpr/Dyn)-treated cells. Fluorescence intensities of ARBM-101 quantified with ImageJ and normalized with Hoechst (B). Data was obtained from 6 independent confocal images from each group. The release of ARBM-101 by exocytosis in HepG2 cells. ARBM-101 co-localization with EEA1, LAMP1, and CD63 in a time-dependent manner (C). Colocalization rates of ARBM-101 with each marker quantified using ZEN 3.5 equipped in the Zeiss confocal microscope. Data was obtained from 10 independent confocal images per group (D). Representative confocal images for the exocytosis of ARBM-101 (E). Fluorescence intensities of ARBM-101 quantified with ImageJ and normalized with Hoechst (F). Immunoblotting and quantification to verify the KD of *ATP7B* and *VAMP3 (G)*. Statistical significance was calculated by one-way ANOVA with Tukey’s method for multiple comparisons. * p ≤ 0.05, ** p ≤ 0.01, **** p ≤ 0.0001.

Hepatic peptide uptake typically involves endosomal processes [28, 29] and biliary copper excretion was demonstrated to occur via the lysosomal-exosomal axis [30]. To obtain mechanistic insights on ARBM-101 driven hepatocytic copper depletion, we investigated these mechanisms using specific inhibitors, to find transcellular routing of ARBM-101, a heavily post-translationally modified peptide [13,18]. Firstly, endocytosis-mediated ARBM-101 uptake into copper-preloaded HepG2 *ATP7B* KD cells was almost fully avoided by either inhibition of clathrin-mediated endocytosis (CME) using chlorpromazine/dynasore (Clpr/Dyn) or, inhibition of macropinocytosis by EIPA (Fig. 1A, quantification in Fig. 1B). Such inhibition was also the case for copper-prebound ARBM-101 (Fig. S1A, quantification in Fig. S1B). Secondly, transcellular ARBM-101 routing via endo-/lysosomal/exocytosis was visualized by its colocalization with EEA1, LAMP1 and CD63, i.e., markers for early endosomes, lysosomes and multivesicular vesicles/exosomes, respectively (Fig. 1C). This analysis revealed a time-dependent increase of colocalization in the latter, coinciding with a decrease in the former (quantification in Fig. 1D, note the partial overlap with two markers), suggesting exocytosis as potential elimination path for ARBM-101. Indeed, thirdly, ARBM-101 was retained in the cells upon depletion of VAMP3, a critical component of exosomal docking to the plasma membrane for exocytosis [31], especially upon removing residual external ARBM-101 by changing to fresh media (Fig. 1E–G). Thus, transcellular routing of ARBM-101 in hepatocytes occurs via endosomal/exosomal trafficking.

### 3.2. Biliary excretion of copper by ARBM-101 in WD rats

**Fig. 2.**
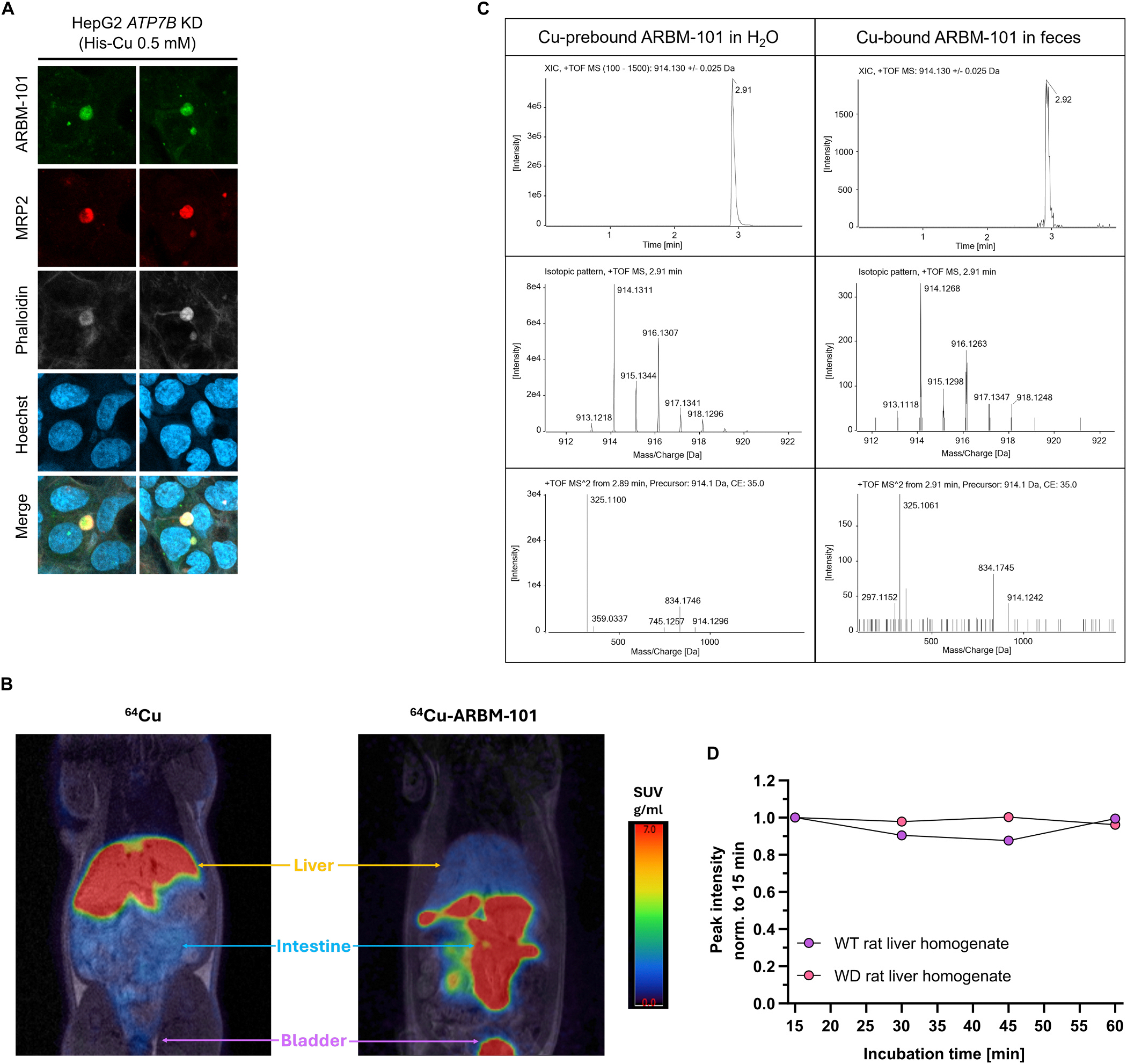
Biliary excretion of copper by ARBM-101. Biliary excretion of internalized ARBM-101 to the bile canaliculus structure *in vitro* (A). The *ATP7B* KD HepG2 cells were maintained on the ECM produced by extended cell culture for 3 days to facilitate the formation of bile structures in vitro. Cells were incubated with 1 mM ARBM-101 for 15 min. MRP2 and phalloidin were used to distinguish the bile structures. ^64^Cu or ARBM-101-prebound ^64^Cu were intravenously injected into WD rats through the tail vein (B). One hour later,^64^Cu radioactivity was monitored by PET/CT for 20 min. Upon injection of ^64^Cu alone (left panel), most of the radioactivity was in the liver, while ARBM-101-prebound ^64^Cu was in the intestinal tract and bladder (right panel). LC-MS/MS spectra of copper-bound ARBM-101 alone or observed in feces from an ARBM-101 treated rat (C). LC-MS/MS data of ARBM-101 incubated with liver homogenates from WT/WD rats (D).

Next, we tested for biliary excretion of ARBM-101 in human WD hepatocyte surrogate cells. HepG2 *ATP7B* KD cells form bile canaliculi-like structures when grown on multi-layered clusters of extracellular matrixes (ECM) [25–27]. Upon cellular treatment for 15 min, ARBM-101 was detected by immunostaining (green fluorescence) in these structures, as evidenced by coinciding red fluorescent MRP2, a biliary/apical surface marker (Fig. 2A). It therefore appears that exosomal transported ARBM-101 is rapidly excreted via this route, thereby possibly allowing for massive copper export. Indeed, *in vivo*, i.e., upon intravenous injection of ^64^Cu prebound to ARBM-101 into WD rats, the ^64^Cu signal was almost immediately detected in the intestine, in stark contrast to injected ^64^Cu alone (Fig. 2B). Moreover, we detected Cu-bound ARBM-101 by LC-MS/MS in 24 h fecal samples of such treated WD rats (Fig. 2C), indicating an only limited metabolization of ARBM-101. Indeed, upon incubation of ARBM-101 with liver homogenate isolated from either WT or WD rats over one hour, no decrease in ARBM-101 peak intensity was encountered by LC-MS/MS (Fig. 2D), confirming absent ARBM-101 metabolization. In first preliminary experiments, this observation was further validated by an incubation of ARBM-101 with kidney homogenate as well as plasma from a WT rat, here also no metabolization of ARBM-101 could be seen within one hour of incubation (Fig. S4).

Thus, ARBM-101 very rapidly passes hepatocytes in a non-metabolized fashion to excrete liver copper via the biliary route into feces.

### 3.3. ARBM-101 depletes excess liver, kidney and plasma copper in severely diseased WD rats

Such rapid liver copper depletion may be of profound therapeutic relevance, especially with respect to acute liver demise that, currently, can only be counteracted by liver transplantation in WD patients. We therefore challenged this hypothesis by aiming to rescue severely diseased WD rats, i.e. animals with a deadly (if untreated) fulminant liver disease phenotype [8,10,11].

**Fig. 3.**
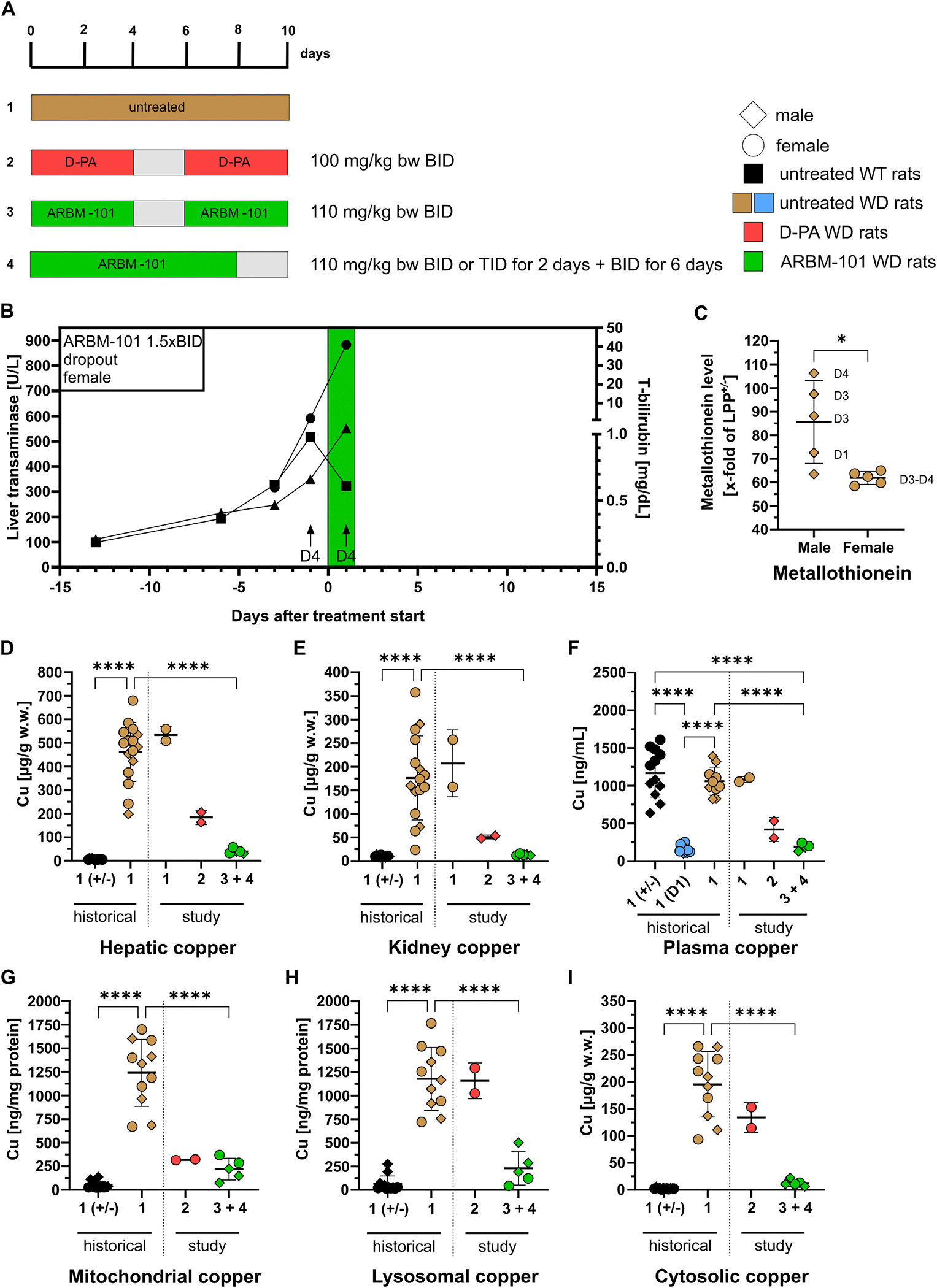
Depletion of excess copper by ARBM-101 in diseased WD rats. Treatment scheme of rat study (A). Change of liver enzymes in female WD rats (B). Metallothionein levels (C) of WD rats. Hepatic copper values (D), kidney copper level (E) and plasma copper level (F) after treatment (day 10). Hepatic mitochondrial (G), lysosomal (H), and cytosolic (I) copper. Historical data served as control. Values with mean ± SD, n = 2–5 per study group. BID: “bis in die” 2 times daily, TID: “ter in die” 3 times daily. Statistical significance was calculated by one-way ANOVA with Tukey’s method for multiple comparisons or by an unpaired *t*-test. Data was not included in the statistical tests if the sample size was 2. * p ≤ 0.05, **** p ≤ 0.0001.

To this, we introduced a fine-tuned scoring system (with disease stages D0 to D4, Table S1) defining the progressive disease states based on the individual but typical pathognomonic features. As described earlier [11], until around 90 days of animal age WD rats are healthy (D0). Disease starts by a stagnation/loss of body weight in these juveniles (one score point) and liver damage becomes progressively apparent by above threshold increased plasma ALT (one score point), AST (one score point) and T-bilirubin (one score point) levels [11]. Thus, upon score point summation, animals at D1/D2 are considered mildly diseased and at D3/D4 diseased/severely diseased. Thus, we asked whether ARBM-101 therapy rescues animals with advanced disease, i.e., at D3/D4 (Fig. 3A).

In our initial experiment, a twice daily ARBM-101 treatment did not rescue a severely diseased female rat that demonstrated a rapid liver decline within one day upon treatment onset (Fig. 3B, Table S2). Conversely, such a treatment for eight days was most effective in diseased male WD rats (see below). This sex-dependent difference was possibly due to lower copper-protective liver metallothionein levels in female vs. male WD rats (Fig. 3C). We thus reasoned that, upon hepatocyte disintegration with a concomitant copper release, neighboring female vs. male hepatocytes would be less copper protected, thereby causing their more rapid demise. This would result in a faster/more pronouncedly propagating “destructive copper wave” in female compared to male WD rat livers. Consequently, considering the rapid ARBM-101 action profile (Figs. 1, 2), we intensified the ARBM-101 treatment regimen, especially in female WD rats, to thrice daily for two days and twice daily for the following six days (Fig. 3A). As summarized in Table S2 and shown below, this intensified regimen was most effective, clearly demonstrating that the driver of liver failure and death in these animals is copper. Indeed, while liver, kidney and plasma copper decreased somewhat by D-PA, the ARBM-101 regimen brought them down to either WT control rat levels (Fig. 3D, E) or, to healthy WD rat levels for plasma (Fig. 3F). Similarly in the mitochondrial, lysosomal, and cytosolic subfractions of the liver, ARBM-101, but not D-PA, reduced the copper burden down to WT level (Fig. 3G–I).

### 3.4. Avoidance of liver failure and full recovery of diseased WD rats by intense ARBM-101 treatments

**Fig. 4.**
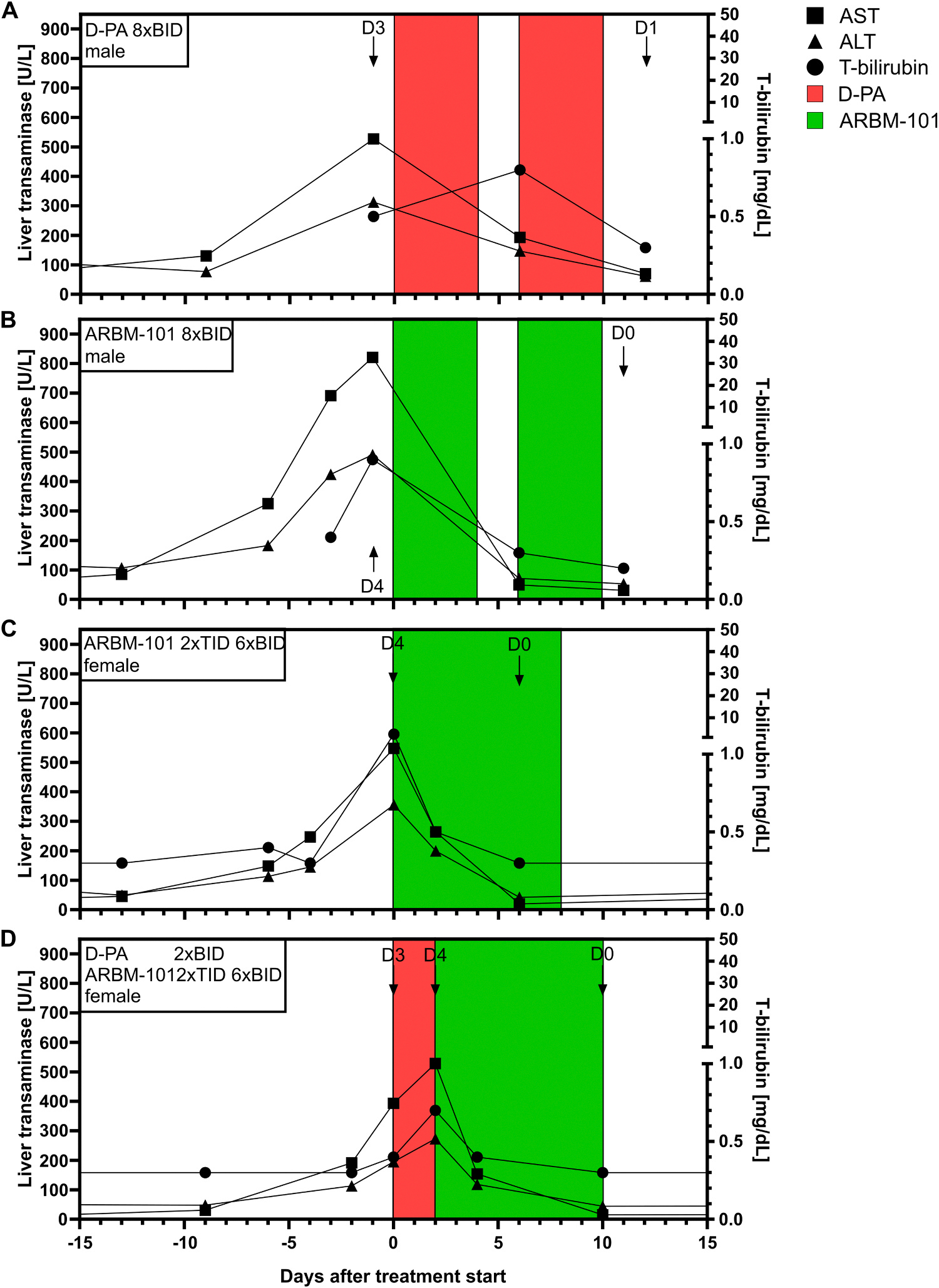
Full recovery of diseased WD rats by intense ARBM-101 treatments. Representative change of liver enzymes (AST and ALT) and T-bilirubin level in plasma of D-PA treated (8xBID) (A), ARBM-101 treated (8xBID) (B), ARBM-101 treated (2xTID + 6xBID) (C) and, D-PA (2xBID) + ARBM-101 (2xTID + 6xBID) (D) WD rats. Red color indicates D-PA treatment period and green color indicates ARBM-101 treatment period. Disease stages (D0-D4) are displayed with arrows.

**Fig. 5.**
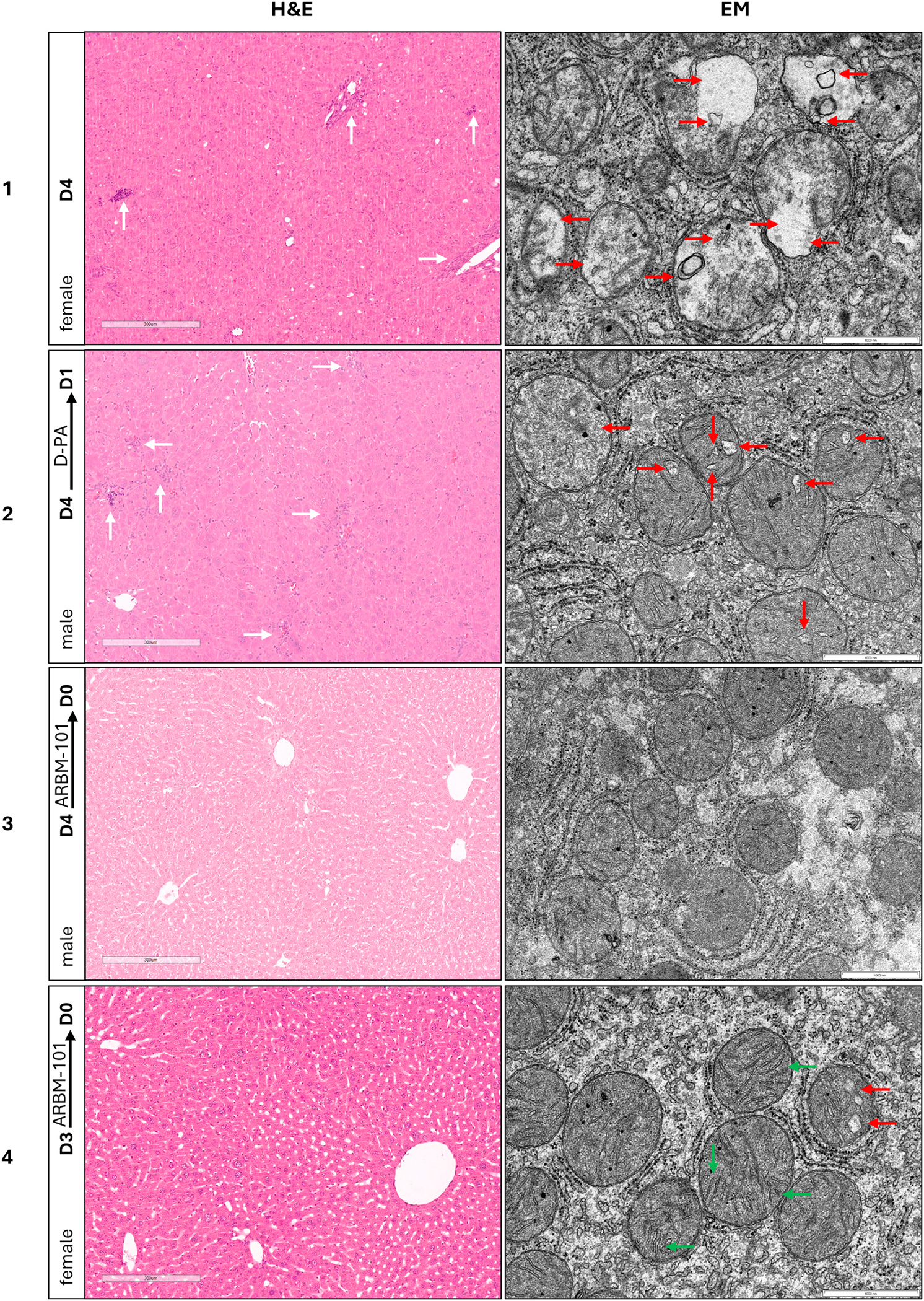
Rescued liver tissue and mitochondria by ARBM-101 in diseased WD rats. Representative H&E-stained formalin-fixed paraffin-embedded liver tissue sections (left) from WD rats at treatment end (Scale bar: 300 μm). White arrows indicate both portal and lobular lymphocytic infiltrates. Representative electron micrographs of liver mitochondria *in situ* (right) (Scale bar: 1000 nm). Impaired liver mitochondrial structures (red arrows) and amelioration of mitochondria structures (more cristae; green arrows).

Fig. 4 exemplarily shows treatment results of diseased/severely diseased female and male WD rats. D-PA improved the disease in male rats (Fig. 4A) from either D2 or D4 to D1 but did not rescue diseased female WD rats (Table S2). However, eight treatment days of twice daily ARBM-101 administration (with intermediary weekend treatment stop), did fully rescue male WD rats from D4 to D0 (Fig. 4B). Importantly, the intensified ARBM-101 regimen also fully rescued diseased female rats from D4 to D0 within one week of treatment (Fig. 4C). D-PA was not effective in such cases, as shown in another female rat even resulting in an increase from D3 to D4 on D-PA. We thus directly switched to intense ARBM-101 treatment in this animal to fully recover it from D4 to D0 (Fig. 4D). In agreement with these therapeutic outcomes, a reduced/absent liver inflammation was noted in D-PA or ARBM-101 treated animals, respectively (Fig. 5 left panels). Moreover, amelioration of liver mitochondrial structures was apparent in D-PA treated WD rats, but almost complete recovery occurred in ARBM-101 treated counterparts (Fig. 5 right panels).

### 3.5. An intense treatment phase by ARBM-101 depletes excess liver copper in WD mice

**Fig. 6.**
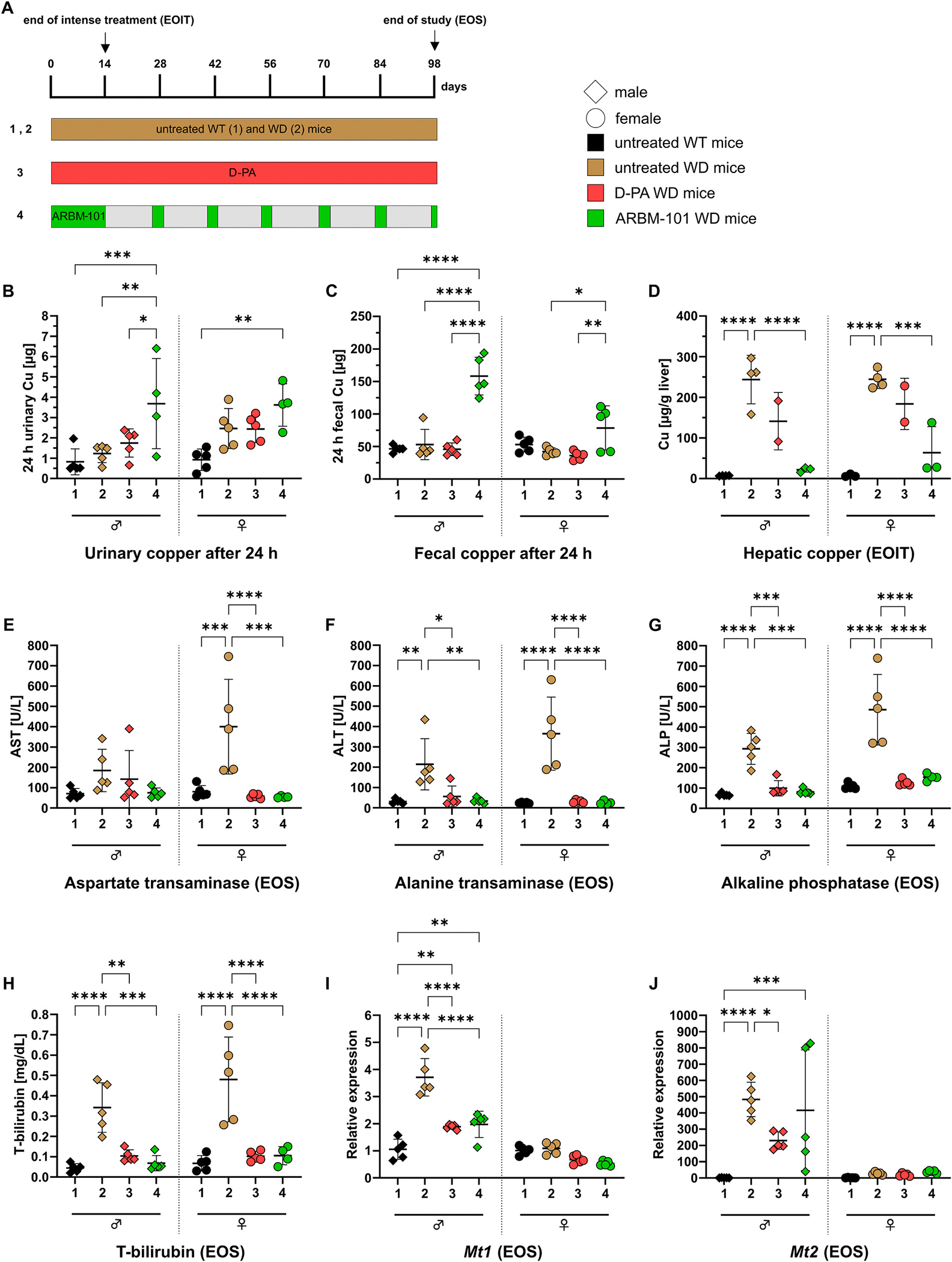
Avoidance of hepatitis by ARBM-101-facilitated copper elimination in WD mice. Treatment scheme of mouse study (A) EOIT = end of intense treatment, EOS = end of study. Urinary copper (B) and fecal copper (C) in males and females after one day of treatment. Hepatic copper values (D) after treatment (EOT, day 14). Aspartate transaminase (AST) (E), alanine transaminase (ALT) (F), alkaline phosphatase (ALP) (G), and T-bilirubin (H) at study end (EOS, day 98). Metallothionein transcript levels for *Mt1* (I) and *Mt2* (J) at study end (EOS, day 98). Values are mean ± SD, n = 5 M/5 F per treatment group, except for: (B) ARBM-101 n = 4 M/4 F; (D) WT n = 4 M/3 F, WD n = 4 M/4 F, D-PA n = 2 M/2 F, ARBM-101 n = 3 M/3 F; and (E-H) ARBM-101 females n = 4. 1 =WT, 2 =untreated WD, 3 =WD+D-PA, 4 = WD+ARBM-101. Statistical significance was calculated by two-way ANOVA with Tukey’s method for multiple comparisons. Data was not included in the statistical tests if the sample size was 2. * p ≤ 0.05, ** p ≤ 0.01, *** p ≤ 0.001, **** p ≤ 0.0001.

To further test ARBM-101 induced liver copper depletion for its long-term perspective and to show its efficacy across species, we treated male and female WD mice (Fig. 6). Urinary and fecal copper excretion and resulting hepatic copper levels in response to D-PA and ARBM-101 treatments were assessed (Fig. 6B–D). Twenty-four hours after treatment start, a tendency for increased urinary copper was observed in male WD mice treated by D-PA that was significant for ARBM-101 (Fig. 6B). However, while excreted urinary copper remained in the single-figure μg range, a drastic fecal copper excretion was initiated by ARBM-101, but not by D-PA (Fig. 6C), in agreement with our earlier findings in WD rats [11]. Furthermore, while urinary copper excretion by D-PA stayed elevated at low absolute level over the treatment period of 14 days, both urinary and fecal copper excretion declined for ARBM-101 towards the end of this treatment (EOIT) (Figs. S2A–H). This decline coincided with a return to almost wildtype (WT) hepatic copper levels in ARBM-101 treated WD mice, whereas a marked but non-significant liver copper depletion was noted for D-PA (Fig. 6D). Thus, as reported in WD rats [11], an intense treatment period by ARBM-101, but not by D-PA, fully depletes excess liver copper into feces in WD mice.

### 3.6. Chelator-treated WD mice do not develop hepatitis and fibrosis

**Fig. 7.**
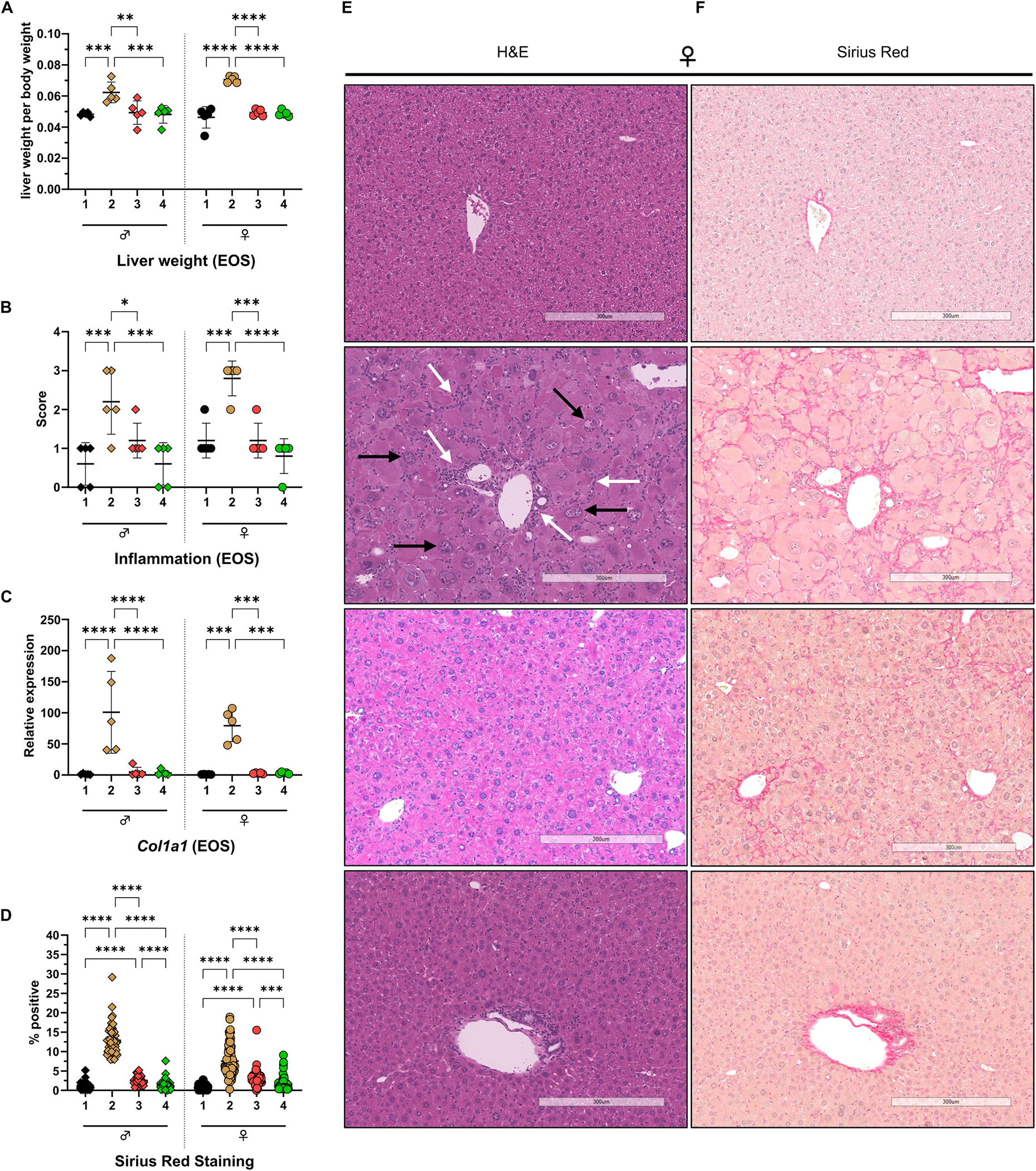
No fibrosis in copper depleted WD mice. Liver weight normalized by body weight (A) at termination (EOS, day 98) after a 14-day treatment period during which ARBM-101 injections were given once a day, every other day, followed by a maintenance injection every 2 weeks until termination. Pathologist score for hepatic inflammation (B) and *Col1a1* (marker of fibrosis) qPCR values (C) and percent of positive Sirius Red staining (D) for ARBM-101 and D-PA treatments at study end (EOS, day 98). Dots were obtained by sampling a 1 × 1 mm tile from each staining slide and further analyzed to recognize Sirius Red stains from background. Values are mean ± SD, n = 5 M/5 F per treatment group. Representative liver sections from female mice at study end, stained with H&E (E) and Sirius Red (F). Scale bar = 300 μm at 10X magnification. Diffusely enlarged and glycogenated hepatocyte nuclei (black arrows) and both portal and lobular lymphocytic infiltrates (white arrows). 1 =WT, 2 =untreated WD, 3 =WD+D-PA, 4 = WD+ARBM-101. Statistical significance was calculated by two-way ANOVA with Tukey’s method for multiple comparisons. * p ≤ 0.05, ** p ≤ 0.01, *** p ≤ 0.001, **** p ≤ 0.0001.

While WD rats develop deadly acute liver failure [8,11], excess copper causes hepatitis and fibrosis in WD mice [32]. We therefore included a prolonged observation period in the treatment scheme of WD mice following intensive alternate day treatments for 14 days. Due to its low copper depletion efficacy, D-PA administration continued in the drinking water, while ARBM-101 was only administered once every second week (Fig. 6A). At the end of the study (EOS, i.e., 182-day-old animals), hepatic copper levels displayed a heterogeneous picture (Fig. S2I). In untreated WD mice, a decrease in EOIT (i.e., after 14 days) vs. EOS (182-day-old animals) was observed that plausibly was due to liver disintegration and copper spill-out (Fig. 6E–H). In D-PA treated mice EOIT and EOS liver copper levels were about the same indicating initial copper depletion at EOIT that, however, balanced out despite continuous treatment (Fig. S2I). Especially in ARBM-101 treated female (but also to some extent in male) WD mice, a re-rise of liver copper occurred between EOIT and EOS (Fig. S2I), indicating that the periodic treatments were insufficient to avoid this re-rise. Importantly, however, the intense initial ARBM-101 treatments ensured a normal body weight gain that was slightly lower in D-PA-treated males, but most prominently aberrant in untreated male WD mice (Fig. S2J). Furthermore, plasma liver enzyme levels AST, ALT, ALP, and T-bilirubin levels were all strongly elevated in untreated WD mice at the end of the study (EOS, i.e., 182-day-old animals) but not in ARBM-101 and D-PA groups, which attained WT levels (Fig. 6E–H). Of note, this was despite increased transcript levels of copper-hepatoprotective metallothioneins [33] *Mt1* and *Mt2* in male but not female WD mice (Fig. 6I–J). Thus, both the continuous D-PA and the intense initial ARBM-101 treatment avoided biochemical signs of liver injury in WD mice at EOS, despite copper re-accumulation associated with ARBM-101 and still elevated copper levels in the D-PA group. This profound therapeutic effect was validated by liver weights (normalized to body weights) that were like WT in D-PA and ARBM-101 treated, but not in untreated WD mice that had significantly higher liver weights than WT in both sexes (Fig. 7A). Most importantly, untreated male and female WD-mouse livers had prominent lobular and portal inflammatory infiltrates and a significantly increased inflammation score with enlarged nuclei at EOS (Figs. 7B, 7E, 7 F, S3). Both treatments, with ARBM-101 being tendentially more efficient, avoided these features (Figs. 7B, 7E, S3). Moreover, *Col1a1* expression, a major biomarker of liver fibrosis [34], and significantly elevated in untreated WD mice, was at WT levels in D-PA and ARBM-101 treated mice (Fig. 7C). This agreed with collagen-reactive Sirius Red staining findings. While untreated WD mice had the most prominent collagen accumulation (Fig. 7 C, 7D, 7 F, S3B), it was significantly less in D-PA treated mice, though still clearly above WT levels (Fig. 7D), whereas fibrosis scores for ARBM-101 treated males and females were not even different from WT (Table S4). Thus, especially the intense initial ARBM-101 treatment avoided inflammation and fibrosis in WD mouse livers until the end of the study.

## 4. Discussion

This study demonstrates that the therapeutic benefit of ARBM-101’s effective liver copper mobilization is due to its “fast-in-fast-out” mechanism via the endosomal/lysosomal/exosomal pathway and its excretion via the biliary/fecal route. This efficacy may be explained by two possibly amplifying reasons. Firstly, the copper affinity of ARBM-101 (KD < 10^−21^ M) [13,14] is orders of magnitude higher than of D-PA (KD = 2.38 × 10^−16^ M) [35]. Indeed, while ARBM-101 can remove copper from metallothionein, D-PA cannot [36] and thus ARBM-101 may not only tightly bind copper from damaged hepatocytes it may also increase the inert copper buffering capacity by reducing the metallothionein copper load. Secondly, as shown by PET studies, ^64^Cu-bound ARBM-101 is excreted within minutes into the gut and mass spectrometry detected it in feces. This clearly argues for a very stable Cu-bound ARBM-101 complex. Indeed, ARBM-101 does not appear to be metabolized by hepatocytes retaining its full de-coppering capacity, and first data validate this absent metabolization in kidney tissue as well.

Compared to further chelators other than D-PA, either in clinical use or under development, these ARBM-101 characteristics indeed are noteworthy. Recently, the 8-aminoquinoline derivative named TDMQ20 has been reported to significantly lower liver copper in WD mice by fecal excretion [37]. Interestingly, at higher doses, TDMQ20 routed a significant Cu amount to the serum and kidneys of treated WD mice, which may need further attention in the future [37]. The preclinical chelator DPM-1001 depletes copper from liver and brain in WD mice, causing elevated fecal copper excretion [38]. However, its comparatively low copper affinity (KD ~75 nM) would predictively not allow for copper removal from metallothionein.

On the other hand, it is important to mention that a very high copper affinity may also cause issues, that is, the potential to remove essential copper, e.g. from mitochondrial cytochrome c oxidase, thereby raising concern about a potential toxic response. This can be seen for Tetra-thiomolybdate that has an enormous copper affinity, but has been reported to cause such toxicity clearly limiting its applied dose [39]. Furthermore, the excretion of Cu-Tetra-thiomolybdate from the body seems to be somewhat limited [39]. It is exactly here where ARBM-101 has an enormous advantage, as Cu- ARBM-101 can already be detected minutes after administration in the gut on its way out via fecal excretion thereby “naturally” limiting potential adverse effects.

Such rapid copper excretion by ARBM-101 enabled most intense, even trice daily, treatment regimen to efficiently treat severely diseased WD rats. In male WD rats, D-PA ameliorated the disease that, nevertheless, was still present at treatment stop. In contrast, ARBM-101 treatments resulted in regained health. In female WD rats, a more rapid decline of liver health status compared to male WD rats was encountered. Plausibly, this is due to a lower level of copper-protective metallothionein that was assessed in female rodents (both WD mice and rats) resulting in a lower hepatocyte capacity to render copper inert. This may come with the drastic consequence that, upon hepatocyte copper overload and disintegration, released copper may hit neighboring hepatocytes more deleteriously in less protected female vs. male livers. A fatal “copper wave” may result that would progress faster and more intense in less protected female vs. male livers. Following these considerations, our rationale was to stop this “copper wave” by more intense treatments. The idea was to most efficiently bind (and safely excrete) both the intrahepatocyte and the therefrom released copper. D-PA, intraperitoneally administered already at a high dosing of 100 mg/kg bw (i.e. 670 μmol/kg bw) precluding further elevation, did not stop female rat liver failure. In contrast, a single ARBM-101 dosing was around five times lower at 110 mg/kg bw (i.e. 130 μmol/kg bw). We therefore increased the typically administered twice daily doses to thrice daily for two days only, whereupon we returned to twice daily for six further days. This intensified ARBM-101 treatment proved to be most effective as all treated female rats at diseased D3/D4 were cured (D0), returning to normal body weights by this regimen. Of note, no “overtreatment” occurred, as cured animals presented with WT copper levels (and not below) in both liver and kidney. Nevertheless, future studies must further substantiate the avoidance of a potentially overshooting copper depletion by ARBM-101. What matters here, however, is that female WD rats, who rapidly worsened despite initiated D-PA treatments, were fully rescued upon direct switching to the intensified ARBM-101 treatment. Thus, in agreement with our supposition, such treatment blocked the “copper wave” and all female animals treated that way were cured, while D-PA failed to do so even at a higher molar dose. Intense (alternate daily) ARBM-101 treatments also depleted excess liver copper in WD mice and avoided long-term complications, i.e. fibrosis development that otherwise is overt in untreated WD mice at 6 months of age. D-PA was of beneficial effect in WD mice concerning lowering liver copper and fibrosis severity as well, however, tested head-to-head, ARBM-101 was markedly more effective.

From the synopsis of these data, it appears that the methanobactin ARBM-101 may be a most beneficial future compound in WD therapy and clinical studies are warrantable. Such therapy would have to rely on i.v. application of this bacteria-derived peptide, where purity, i.e. absent other bacteria-originated contaminants are essential. Indeed, neither in this study nor in earlier ones [10,11] adverse reactivities like a potential toxicity or immunogenicity of ARBM-101 were encountered, especially not in a recently reported long-term study in WD rats [11]. Nevertheless, such potential safety concerns are key issues with respect to future clinical studies and therefore will remain in research focus. One particularly interesting feature of ARBM-101 is its versatility with respect to dosing. In an earlier study, we showed that this compound dose-dependently depleted liver copper parallel to concomitant healthy periods [11]. Here we have taken full advantage of this feature by applying ARBM-101 thrice daily in severely diseased animals to enforce massive copper excretion and block liver disintegration. It remains to be seen, however, whether such a strategy would be applicable in a human setting also involving potential resistance mechanisms.

## 5. Conclusions

In conclusion, we report here the rapid transcellular hepatocyte crossing of ARBM-101, allowing for most efficient liver copper mobilization and excretion into bile and feces in WD rats and WD mice. This enables safe rescue from severely damaged liver stages and avoids fibrosis development. Moreover, the diverse dosing applied in this study without obvious signs of toxicity, may possibly allow for treatments adapted to the individual WD patients in the future.

## Supplementary Material

1

## Figures and Tables

**Table 1:** Primer sequences for *Col1a1*, *Mt1*, and *Mt2*.

Gene		Primer	Sequence 5′ to 3′	Exon-exon spanning	Amplicon	% Primer efficiency

*Col1a1*	Collagen Type 1 alpha 1	F	ACGGCTGCACGAGTCACAC	Yes	132	105.6
		R	CTAGTCCGAATTCCTGGTCTGG	No		
*Mt1*	Metallothionein 1	F	GCTGTCCTCTAAGCGTCACCA	No	97	108.2
		R	TGGGGTCCATTCCGAGATCTGG	No		
*Mt2*	Metallothionein 2	F	CAAACCGATCTCTCGTCGAT	No	150	102.1
		R	AGGAGCAGCAGCTTTTCTTG	Yes		

## Data Availability

All data are available in the main text or the supplementary material.

## Appendix A. Supporting information

Supplementary data associated with this article can be found in the online version at doi:10.1016/j.biopha.2025.118867.
