# Supplementary material for: Rapid transcellular hepatic copper depletion by ARBM-101 rescues severe liver damage in Wilson disease rodents
Source: Biomed Pharmacother. Author manuscript; Available in PMC 2026 Jul 17. (PMC13378520; doi:10.1016/j.biopha.2025.118867)
Supplement: 1 [file NIHMS2187305-supplement-1.docx]

**SUPPLEMENTARY MATERIAL**

Fig. S1: Intracellular imaging of ARBM-101 in hepatocytes.

Fig. S2: Excretion of copper in WD mice upon ARBM-101 treatment.

Fig. S3: Reduction of signs of damage in WD mice after ARBM-101 treatment.

Fig. S4: No metabolization of ARBM-101 in kidney or plasma of WT rats.

Table S1. Disease scoring parameters of WD rats.

Table S2. Disease scores of WD rats.

Table S3. Food/water intake of WD mice.

Table S4. Fibrosis score of WD mice.


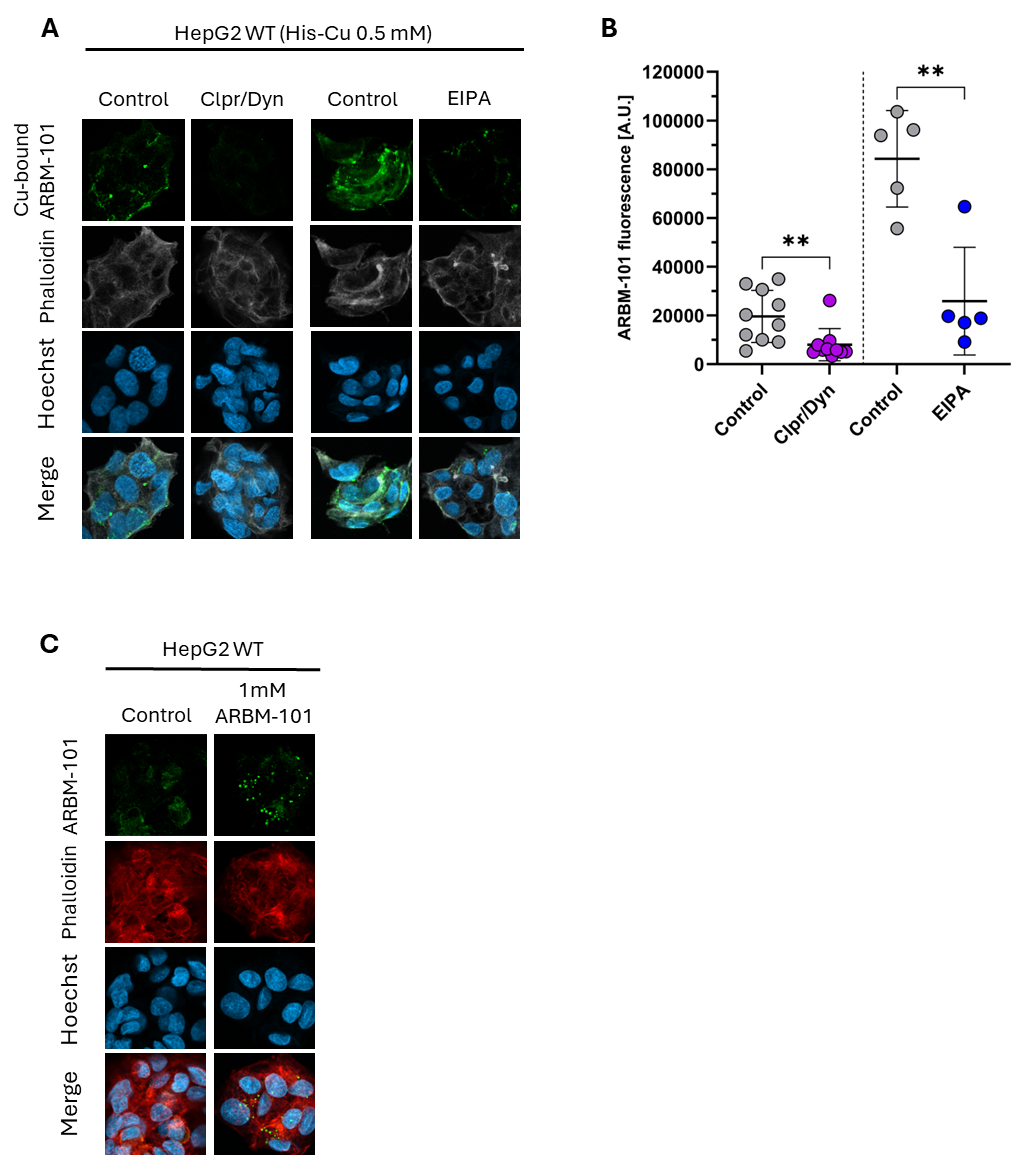


**Fig. S1: Intracellular imaging of ARBM-101 in hepatocytes.**

Representative confocal images of macropinocytosis and CME of Cu-prebound ARBM-101 in WT HepG2 cells (A). The intracellular fluorescent signal of ARBM-101 was detected in untreated control cells, and EIPA-treated cells and, Chlorpromazine-Dynasore(Clpr/Dyn)-treated cells. Fluorescence intensities of ARBM-101 quantified with ImageJ and normalized with Hoechst (B). Data was obtained from 6 independent confocal images from each group. Representative confocal images of R1 antibody specificity (C). Statistical significance was calculated by one-way ANOVA with Tukey’s method for multiple comparisons. ** p ≤ 0.01.

**
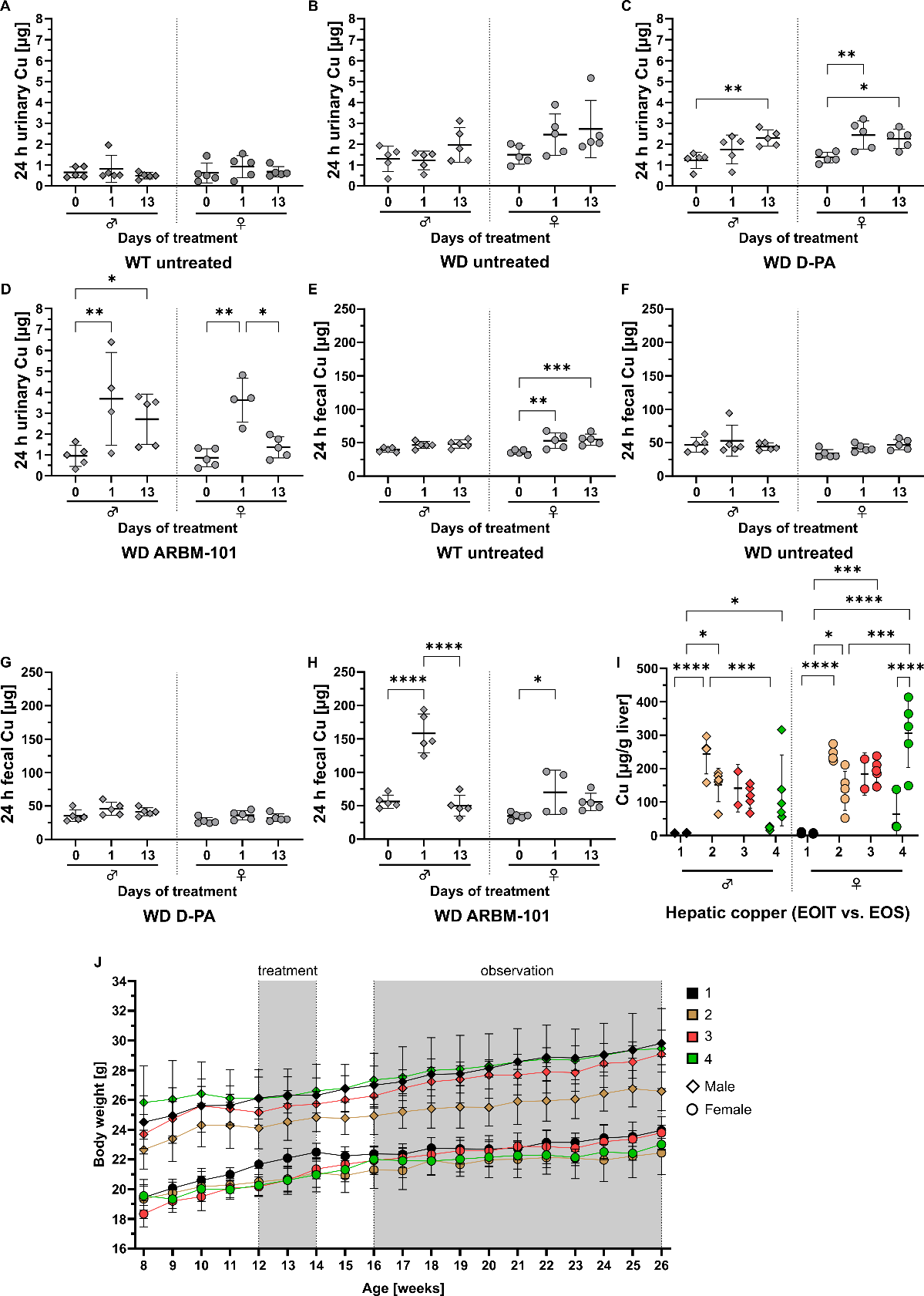
**

**Fig. S2: Excretion of copper in WD mice upon ARBM-101 treatment.**

Urinary (A-D) and fecal (E-H) copper in male and female mice before treatment, after one day of treatment and, at treatment end (EOIT). Untreated WT mice (A, E), untreated WD mice (B, F), D-PA treated WD mice (C, G) and, ARBM-101 treated WD mice (D, H). Hepatic copper values (I) at intense treatment end and study end (EOIT, day 14 vs. EOS, day 98). Body weight during study (J). 1=WT, 2=untreated WD, 3=WD+D-PA, 4= WD+ARBM-101. Statistical significance was calculated by two-way ANOVA with Tukey’s method for multiple comparisons. * p ≤ 0.05, ** p ≤ 0.01, *** p ≤ 0.001, **** p ≤ 0.0001.


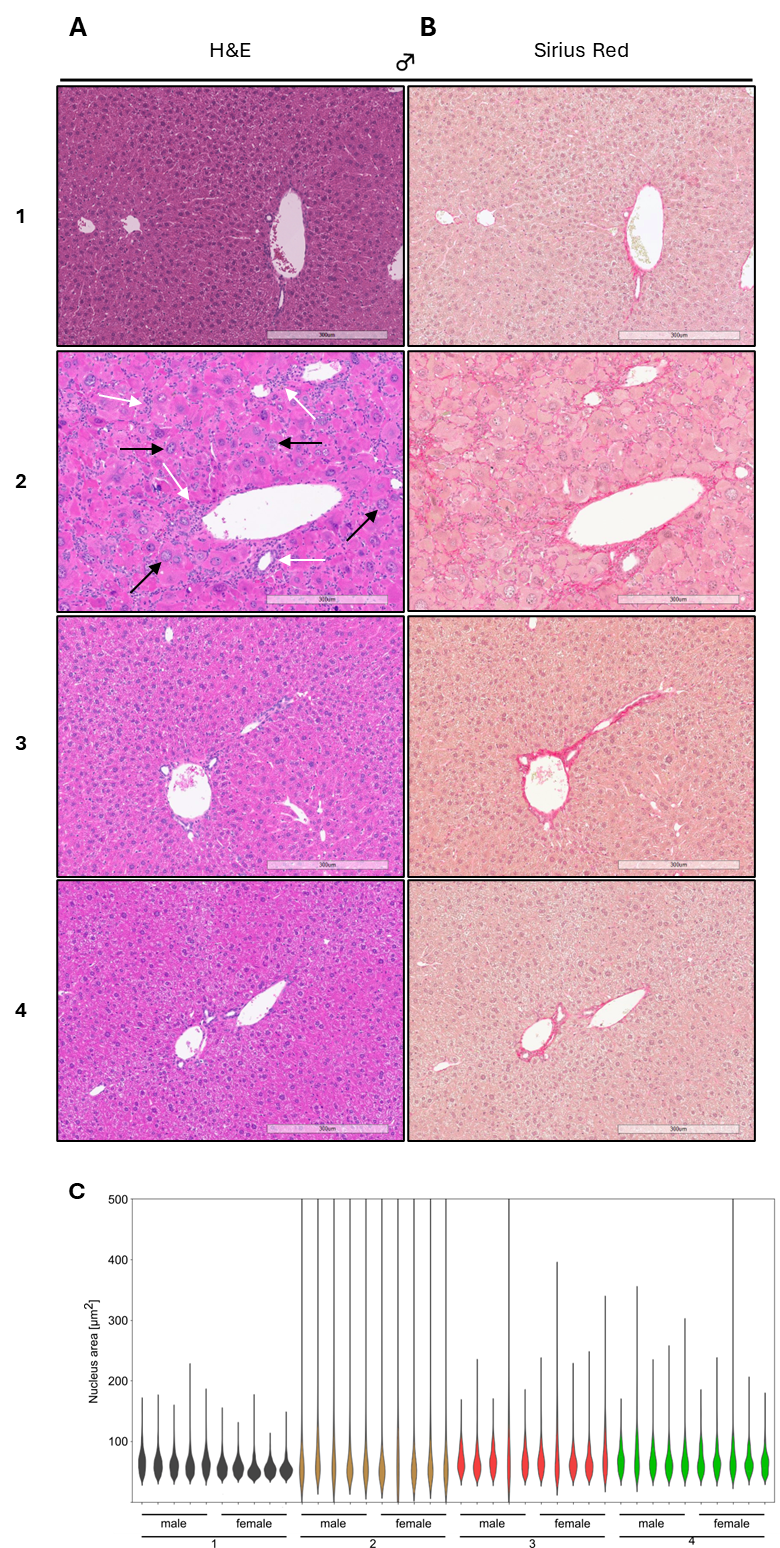


**Fig. S3: Reduction of signs of damage in WD mice after ARBM-101 treatment.**

Representative liver sections from male mice at study end, stained with H&E (A) and Sirius Red (B). Scale bar = 300 μm at 10X magnification. Liver histology is normal in WT mice. Untreated WD mice livers presented diffusely enlarged and glycogenated hepatocyte nuclei (black arrows) and both portal and lobular lymphocytic infiltrates (white arrows) with an average fibrosis score of 2.1 ± 0.2. Nucleus size (C) at termination (EOS, day 98) after a 14-day treatment period during which ARBM-101 injections were given once a day, every other day, followed by a maintenance injection every 2 weeks until termination. Values are displayed in violin plot, n = 5M/5F per treatment group. 1=WT, 2=untreated WD, 3=WD+D-PA, 4= WD+ARBM-101.


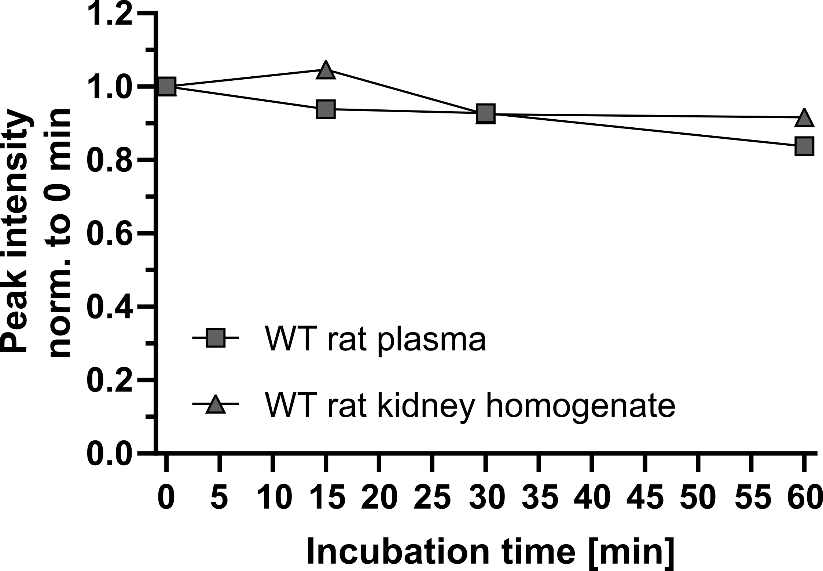


**Fig. S4: No metabolization of ARBM-101 in kidney or plasma of WT rats.**

LC-MS/MS data of ARBM-101 incubated with kidney homogenates or plasma from WT rats. Preliminary data with n=1.

**Table S1.** **Disease scoring parameters of WD rats**. Criteria for scoring disease progression and therapy efficacy in WD rats.

| **Disease Score** | **Summary score** |
| --- | --- |
| D0 | 0 |
| D1 | 1 |
| D2 | 2 |
| D3 | 3 |
| D4 | 4 |
|  | |
| **Factor** | **Score point** |
| ALT ≥100 U/L | 1 |
| AST ≥200 U/L | 1 |
| T-BIL ≥0.4 mg/dL | 1 |
| Body weight loss ≥1% | 1 |

**Table S2.** **Disease scores of WD rats**. Disease score of WD rats before and after treatment.

| Group | Genotype | Sex | Treatment | Score at start | Score at end |
| --- | --- | --- | --- | --- | --- |
| 1 | -/- | female | untreated | D4 | - |
|  | -/- | female | untreated | D4 | - |
| 2 | -/- | male | D-PA 8xBID | D4 | D1 |
|  | -/- | male | D-PA 8xBID | D2 | D1 |
| 3 | -/- | male | ARBM-101 8xBID | D4 | D0 |
|  | -/- | male | ARBM-101 8xBID | D4 | D0 |
|  | -/- | female | ARBM-101 8xBID | D1 | D0 |
|  | -/- | female | ARBM-101 8xBID | D2 | D0 |
|  | -/- | female | ARBM-101 2xTID and 6xBID | D3 | D0 |
| 4 | -/- | male | ARBM-101 2xTID and 6xBID | D4 | D0 |
|  | -/- | female | ARBM-101 2xTID and 6xBID | D4 | D0 |
|  | -/- | female | D-PA 2xBID 🡪 ARBM-101 2xTID and 6xBID | D3 | D0 |
|  | -/- | female | D-PA 2xBID 🡪 ARBM-101 2xTID and 6xBID | D4 | D0 |
| therapy failure | -/- | female | D-PA, 5xBID | D2 | D4 |
|  | -/- | female | D-PA, 3xBID | D3 | D4 |
|  | -/- | female | ARBM-101, 1.5xBID | D4 | D4 |
|  | -/- | female | ARBM-101, 1xBID | D4 | D4 |

**Table S3.** **Food/water intake of WD mice.** Food and water intake remained relatively unchanged among most groups during treatment and observation periods, except for D-PA males and females where food intake was reduced and remained lower after treatment began.

| Genotype | Sex | Treatment | Dose  [mg/kg bw] | Day 0 | | Day 1 | | Day 5 | | Day 98 | |
| --- | --- | --- | --- | --- | --- | --- | --- | --- | --- | --- | --- |
|  |  |  |  | Food [g] | Water [mL] | Food [g] | Water [mL] | Food [g] | Water [mL] | Food [g] | Water [mL] |
| WT | M | no treatment | - | 7.2 ± 2.3 | 6.7 ± 2.2 | 5.5 ± 1.5 | 5.9 ± 1.7 | 5.1 ± 1.4 | 5.8 ± 1.8 | 5.5 ± 1.5 | 6.1 ± 1.9 |
| WT | F | no treatment | - | 7.3 ± 2.4 | 6.9 ± 2.3 | 5.1 ± 1.3 | 6.3 ± 2.0 | 5.1 ± 1.4 | 6.6 ± 2.2 | 5.5 ± 1.5 | 6.9 ± 2.3 |
| KO | M | no treatment | - | 7.2 ± 2.3 | 6.7 ± 2.2 | 5.2 ± 1.3 | 5.1 ± 1.5 | 4.8 ± 1.2 | 5.2 ± 1.5 | 5.0 ± 1.3 | 4.4 ± 1.2 |
| KO | F | no treatment | - | 7.1 ± 2.3 | 6.8 ± 2.2 | 5.0 ± 1.4 | 7.1 ± 2.7 | 5.1 ± 1.3 | 7.9 ± 3.1 | 5.5 ± 1.6 | 7.5 ± 2.7 |
| KO | M | D-PA | 100 | 7.4 ± 2.5 | 6.2 ± 1.9 | 4.3 ± 0.9 | 5.3 ± 1.5 | 4.1 ± 0.9 | 5.3 ±1.4 | 4.5 ± 1.0 | 5.8 ± 1.7 |
| KO | F | D-PA | 100 | 7.2 ± 2.3 | 6.4 ± 2.3 | 3.9 ± 0.8 | 5.5 ± 1.6 | 3.5 ± 0.6 | 5.7 ± 1.6 | 3.9 ± 0.8 | 5.8 ± 1.6 |
| KO | M | ARBM-101 | 200 | 7.4 ± 2.4 | 7.0 ± 2.5 | 5.2 ± 1.4 | 6.9 ± 2.7 | 5.5 ± 1.5 | 6.3 ± 2.2 | 5.4 ± 1.5 | 6.6 ± 2.7 |
| KO | F | ARBM-101 | 200 | 7.2 ± 2.3 | 6.2 ± 2.3 | 5.0 ± 1.3 | 5.5 ± 1.6 | 5.1 ± 1.3 | 6.2 ± 1.9 | 5.2 ± 1.4 | 6.3 ± 2.0 |

**Table S4.** **Fibrosis score of WD mice**. Fibrosis scores for ARBM-101 males and females were not different from WT. While fibrosis in D-PA mice was significantly less than untreated, it was still significantly increased compared with WT males. There were no other significant differences between ARBM-101 and WT in male and female mice.

| Genotype | Sex | Treatment | Dose  [mg/kg bw] | Fibrosis  Score |
| --- | --- | --- | --- | --- |
| WT | M | no treatment | - | 1.0 |
| WT | M | no treatment | - | 1.0 |
| WT | M | no treatment | - | 0.5 |
| WT | M | no treatment | - | 0.5 |
| WT | M | no treatment | - | 0.5 |
| WT | F | no treatment | - | 1.0 |
| WT | F | no treatment |  | 1.0 |
| WT | F | no treatment | - | 1.0 |
| WT | F | no treatment | - | 1.0 |
| WT | F | no treatment | - | 1.0 |
| KO | M | no treatment | - | 2.0 |
| KO | M | no treatment | - | 2.0 |
| KO | M | no treatment | - | 2.5 |
| KO | M | no treatment | - | 2.0 |
| KO | M | no treatment | - | 2.0 |
| KO | F | no treatment | - | 2.5 |
| KO | F | no treatment | - | 2.0 |
| KO | F | no treatment | - | 2.5 |
| KO | F | no treatment | - | 2.0 |
| KO | F | no treatment | - | 2.5 |
| KO | M | D-PA | 100 | 1.5 |
| KO | M | D-PA | 100 | 1.5 |
| KO | M | D-PA | 100 | 1.0 |
| KO | M | D-PA | 100 | 1.5 |
| KO | M | D-PA | 100 | 1.5 |
| KO | F | D-PA | 100 | 1.0 |
| KO | F | D-PA | 100 | 2.0 |
| KO | F | D-PA | 100 | 1.0 |
| KO | F | D-PA | 100 | 1.0 |
| KO | F | D-PA | 100 | 1.0 |
| KO | M | ARBM-101 | 200 | 0.5 |
| KO | M | ARBM-101 | 200 | 2.0 |
| KO | M | ARBM-101 | 200 | 1.0 |
| KO | M | ARBM-101 | 200 | 0.5 |
| KO | M | ARBM-101 | 200 | 1.0 |
| KO | F | ARBM-101 | 200 | 1.0 |
| KO | F | ARBM-101 | 200 | 1.0 |
| KO | F | ARBM-101 | 200 | 1.0 |
| KO | F | ARBM-101 | 200 | 0.5 |
| KO | F | ARBM-101 | 200 | 1.0 |
